# Hantavirus-infection Confers Resistance to Cytotoxic Lymphocyte-Mediated Apoptosis

**DOI:** 10.1371/journal.ppat.1003272

**Published:** 2013-03-28

**Authors:** Shawon Gupta, Monika Braun, Nicole D. Tischler, Malin Stoltz, Karin B. Sundström, Niklas K. Björkström, Hans-Gustaf Ljunggren, Jonas Klingström

**Affiliations:** 1 Department of Microbiology, Tumor and Cell Biology, Karolinska Institutet, Stockholm, Sweden; 2 Department of Preparedness, Swedish Institute for Communicable Disease Control, Solna, Sweden; 3 Center for Infectious Medicine, Department of Medicine, Karolinska Institutet, Karolinska University Hospital Huddinge, Stockholm, Sweden; 4 Fundación Ciencia & Vida, Santiago, Chile; 5 Liver Immunology Laboratory, Division of Gastroenterology and Hepatology, Department of Medicine, Karolinska Institutet, Karolinska University Hospital Huddinge, Stockholm, Sweden; University of North Carolina at Chapel Hill, United States of America

## Abstract

Hantaviruses cause hemorrhagic fever with renal syndrome (HFRS) and hantavirus cardio-pulmonary syndrome (HCPS; also called hantavirus pulmonary syndrome (HPS)), both human diseases with high case-fatality rates. Endothelial cells are the main targets for hantaviruses. An intriguing observation in patients with HFRS and HCPS is that on one hand the virus infection leads to strong activation of CD8 T cells and NK cells, on the other hand no obvious destruction of infected endothelial cells is observed. Here, we provide an explanation for this dichotomy by showing that hantavirus-infected endothelial cells are protected from cytotoxic lymphocyte-mediated induction of apoptosis. When dissecting potential mechanisms behind this phenomenon, we discovered that the hantavirus nucleocapsid protein inhibits the enzymatic activity of both granzyme B and caspase 3. This provides a tentative explanation for the hantavirus-mediated block of cytotoxic granule-mediated apoptosis-induction, and hence the protection of infected cells from cytotoxic lymphocytes. These findings may explain why infected endothelial cells in hantavirus-infected patients are not destroyed by the strong cytotoxic lymphocyte response.

## Author Summary

Rodent-born hantaviruses cause two severe emerging diseases with high case-fatality rates in humans; hemorrhagic fever with renal syndrome (HFRS) in Eurasia and hantavirus cardio-pulmonary syndrome (HCPS; also called hantavirus pulmonary syndrome (HPS)) in the Americas. A hallmark of HFRS/HCPS is increased vascular permeability. While endothelial cells are the main targets for hantaviruses, infection *per se* is not lytic. Patients suffering from HFRS and HCPS show remarkable strong cytotoxic lymphocyte responses including high numbers of activated NK cells and antigen-specific CD8 T cells. Hence, it has been suggested that cytotoxic lymphocyte-mediated killing of hantavirus-infected endothelial cells might contribute to HFRS/HCPS-pathogenesis. Here, we show that hantaviruses protect infected endothelial cells from being killed by cytotoxic lymphocytes. Further, we also show that hantaviruses inhibit apoptosis in general. Hantaviruses are negative-stranded RNA viruses encoding four structural proteins. Interestingly, the nucleocapsid protein was shown to inhibit the enzymatic functions of both granzyme B and caspase 3, two enzymes crucial for cytotoxic lymphocyte-mediated killing of virus-infected cells. Our study provides new insights into the interactions between hantaviruses, infected cells, and cytotoxic lymphocytes, and argues against a role for cytotoxic lymphocyte-mediated killing of virus-infected endothelial cells in causing HFRS/HCPS.

## Introduction

Hantaviruses are emerging zoonotic viruses that cause two severe diseases: hemorrhagic fever with renal syndrome (HFRS) in Eurasia and hantavirus cardio-pulmonary syndrome (HCPS; also called hantavirus pulmonary syndrome (HPS)) in the Americas, with case-fatality rates of up to 10% for HFRS and up to 40% for HCPS [1], [2]. Several different hantaviruses cause HFRS and HCPS. Among them, Hantaan virus (HTNV) and Andes virus (ANDV) are the most common HFRS- and HCPS-causing hantaviruses, respectively [1], [2]. Endothelial cells are the main targets for hantaviruses and increased vascular permeability is, as in other hemorrhagic fevers [3], a hallmark of HFRS and HCPS. The underlying mechanisms of the increased vascular permeability observed in HFRS and HCPS are, however, not completely understood. For example, it is unclear whether hantaviruses themselves or, alternatively, the related immune responses, are responsible for causing pathology [1], [4]–[6].

Strong CD8 T cell responses are observed in hantavirus-infected patients [7], [8]. Recent data have also demonstrated that HFRS-patients exhibit a rapid expansion of activated natural killer (NK) cells that in many patients persist at elevated numbers for a prolonged period of time [9]. However, autopsies performed on deceased patients have not revealed any obvious damage of hantavirus-infected endothelial cells [4], [10]–[12]. This contradiction suggests that hantaviruses might possess mechanisms to prevent cytotoxic lymphocytes from killing infected endothelial cells. This reasoning led us to address how hantavirus-infected endothelial cells are affected by cytotoxic lymphocytes.

The cytotoxic granule-dependent pathway, involving granzyme B-mediated activation of caspase 3, is the main pathway used by cytotoxic lymphocytes, including CD8 T cells and NK cells, to induce apoptosis in virus-infected cells [13], [14]. Here, we show that hantavirus-infected endothelial cells survive exposure to NK cells, cytotoxic lymphocytes preloaded with large amounts of perforin and granzymes [15]. In an analysis of possible mechanisms behind this phenomenon, the hantavirus nucleocapsid protein was found to inhibit the function of granzyme B and caspase 3.

## Results

### Hantavirus-infected cells are protected from cytotoxic lymphocyte-mediated apoptosis

**Figure 1:**
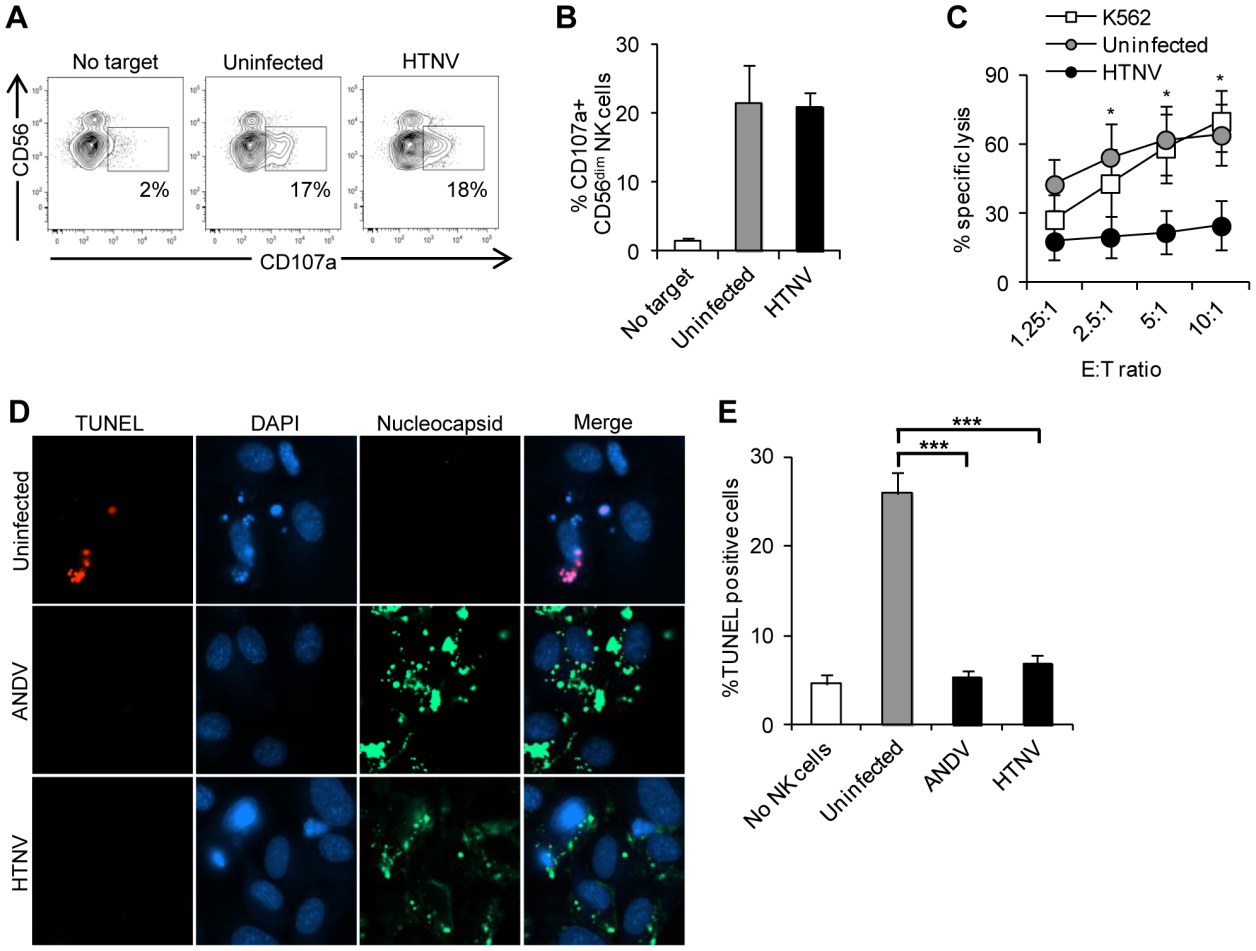
Hantaviruses inhibit NK cell-mediated killing of infected endothelial cells. Endothelial cells were infected with hantavirus for three days or left uninfected (control). Infected and uninfected cells were then exposed to IL-2-activated NK cells. (**A**) Representative flow cytometry plots showing CD107a-expression on IL-2-activated CD56^dim^ NK cells either not exposed to target cells or two hours after exposure to uninfected or HTNV-infected endothelial cells. (**B**) Frequencies of CD107a-positive CD56^dim^ NK cells after co-culture with uninfected or HTNV-infected endothelial cells quantified using flow cytometry. Data shown represent mean ± standard error of mean (SEM) from three donors. One-way ANOVA was used for statistical evaluation: there was no significant difference between NK cells exposed to uninfected versus HTNV-infected endothelial cells. (**C**) Frequencies of killed target endothelial cells after exposure to different ratios of NK cells. NK cells and uninfected or HTNV-infected endothelial cells were co-incubated for four hours, followed by quantification of killed target endothelial cells by flow cytometry. Data shown represent the mean ± SEM of two independent experiments from five donors. K562 cells were used as a positive control for NK cell killing. Two-way ANOVA was used for statistical evaluation; * p<0.05. E∶T ratio; effector to target ratio. (**D**) Representative immuno-fluorescence images of uninfected, ANDV-infected and HTNV-infected endothelial cells after two hours of co-incubation with NK cells. Cells were stained with TUNEL (red) to show apoptosis, mAbs 7B3/F7 for ANDV (green) or 7A2/D5 for HTNV (green) to detect hantavirus nucleocapsid protein in infected cells, and DAPI (blue) for nuclear staining. Images are representative data from five donors. (**E**) Percentages of TUNEL-positive uninfected and ANDV-, or HTNV-infected endothelial cells after co-incubation with NK cells at a 1∶1 ratio for two hours. TUNEL-positive cells were identified using fluorescence microscopy. Data shown represent the mean ± SEM of two independent experiments from five donors. One-way ANOVA was used for statistical evaluation; *** p<0.001.

To study the responses of cytotoxic lymphocytes upon recognition of hantavirus-infected endothelial cells, HTNV-infected and uninfected primary endothelial cells were exposed to peripheral blood-derived short-term IL-2 activated NK cells. HLA class I molecules, ligands for NK cell-inhibitory receptors [16], were blocked on the infected and uninfected endothelial cells to allow for maximal NK cell-responses. First, NK cell-degranulation towards infected and uninfected endothelial cells was assessed by measurement of surface expression of CD107a, a surrogate marker for lymphocyte degranulation [17]. Similar levels of degranulation were observed for NK cells co-incubated with infected and uninfected endothelial cells (Figures 1A–B), suggesting that the target cells were exposed to similar levels of cytolytic granule content. This prompted us to study the effects of NK cell interaction with the endothelial cells directly. Strikingly, while uninfected endothelial cells were killed, infected endothelial cells survived (Figure 1C). This indicated to us that hantavirus-infected endothelial cells might be protected from NK cell-mediated induction of apoptosis. In support of this notion, exposure of infected endothelial cells to NK cells resulted in virtually no signs of apoptosis assessed by TUNEL-staining, while a marked induction of apoptosis was apparent in uninfected endothelial cells under the same conditions (Figures 1D–E). Although surprising, the present findings corroborated earlier findings showing that hantavirus-infected endothelial cells in patient material, despite strong cytotoxic lymphocyte responses, are generally not damaged [4], [7]–[12].

### Hantavirus-infection inhibits staurosporine-induced apoptosis

**Figure 2:**
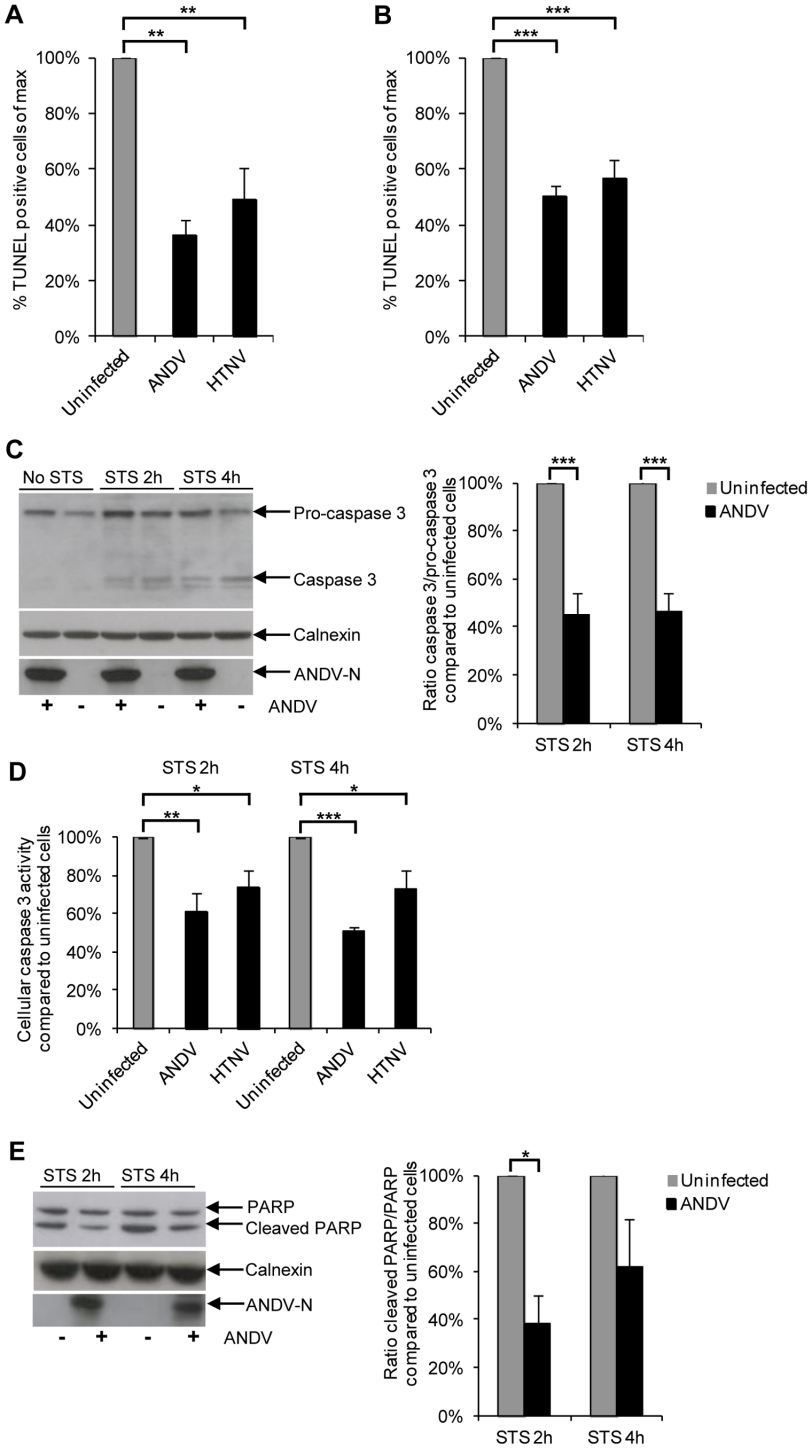
Hantaviruses inhibit staurosporine-induced apoptosis. Endothelial and epithelial cells were infected with hantavirus for three days or left uninfected (control). Cells were then exposed to the apoptosis-inducing drug staurosporine, and subsequently analyzed for induction of apoptosis. (**A and B**) Percentage of TUNEL-positive endothelial cells (A) and A549 cells (B) infected with ANDV or HTNV for three days and then treated with staurosporine for four hours. A low level of ANDV or HTNV (MOI 0.01 for both) was used for initial infection to allow for a level of approximately 20% infected cells at three days pi, the time at which cells were exposed to staurosporine. Amounts of TUNEL-positive infected and uninfected cells on the same slides were then determined using fluorescence microscopy. Staurosporine-treated uninfected cells represent maximal level of TUNEL-positive cells. Data shown represent the mean ± SEM of three independent experiments carried out in duplicate. One-way ANOVA was used for statistical evaluation; ** p<0.01; *** p<0.001. (**C**) Western blot analyses of pro-caspase 3 and caspase 3 from lysate of staurosporine-treated ANDV-infected and uninfected A549 cells. ANDV nucleocapsid protein (ANDV-N) was visualized with the mAb 7B3/F7. Calnexin was used as a control to show that similar amounts of protein were loaded in all wells. One representative experiment out of three is shown. Band intensity was analyzed by densitometry of cleaved, active, caspase 3 and full-length, inactive, pro-caspase 3. The caspase 3/pro-caspase 3-ratio was then calculated and compared between infected and uninfected cells. Staurosporine-treated uninfected cells represent the maximal caspase 3/pro-caspase 3 ratio at the indicated time-points after start of staurosporine-treatment. Data shown represent the mean ± SEM of three independent experiments. Two-way ANOVA was used for statistical evaluation; *** p<0.001. STS; staurosporine. (**D**) Caspase 3-activity after staurosporine-treatment of ANDV or HTNV infected A549 cells compared to that detected in uninfected A549 cells at the same time points. Levels of total protein in the samples were measured using a Bradford assay. Staurosporine-treated uninfected cells represent maximal level of caspase 3-activity/mg of total cellular protein. Data shown represent the mean ± SEM of three independent experiments carried out in duplicate. Two-way ANOVA was used for statistical evaluation; * p<0.05; ** p<0.01; *** p<0.001. STS; staurosporine. (**E**) Western blot analyses of full-length PARP and of caspase 3-cleaved PARP in staurosporine-treated uninfected and ANDV-infected A549 cells. ANDV-N was visualized with the mAb 7B3/F7. Calnexin was used as a control to show that similar amounts of protein were loaded in all wells. One representative experiment out of three is shown. Band intensity was analyzed by densitometry of caspase 3-cleaved PARP and full-length PARP. The caspase 3-cleaved PARP/PARP-ratio was then calculated and compared between infected and uninfected cells. Staurosporine-treated uninfected cells represent maximal caspase 3-cleaved PARP/full-length PARP-ratio at the indicated time-points after start of staurosporine-treatment. Data shown represent the mean ± SEM of three independent experiments. Two-way ANOVA was used for statistical evaluation; * p<0.05. STS; staurosporine.

The finding that hantaviruses inhibited cytotoxic lymphocyte-mediated cell death downstream of degranulation suggested the possibility that these viruses interfere with induction of apoptosis in infected cells. This led us to first test if hantaviruses could inhibit apoptosis in general, i.e., not only specifically induced by cytotoxic lymphocytes. Staurosporine is a broad kinase-inhibitor that activates several different apoptosis-inducing pathways [18], [19]. Endothelial cells infected with ANDV or HTNV were thus exposed to staurosporine and then monitored for apoptosis. Compared to uninfected cells, both ANDV-infected and HTNV-infected cells showed clearly lower levels of apoptosis (Figure 2A). To test if hantavirus-mediated inhibition of staurosporine-induced apoptosis could be observed also in cell types other than endothelial cells, we examined the effects of staurosporine-induced apoptosis in the human lung epithelial cell line A549. Likewise, both ANDV- and HTNV-infected epithelial cells showed clearly lower levels of apoptosis compared to uninfected cells (Figure 2B). This suggests that hantavirus-mediated inhibition of apoptosis is not limited to cytotoxic lymphocyte-mediated apoptosis, nor is it restricted to endothelial cells.

Induction of apoptosis by the cytotoxic granule-dependent pathway and by staurosporine eventually converges into cleavage of pro-caspase 3 into active caspase 3 [14], [19], which in turn cleaves cellular proteins leading to apoptosis. The cleaved, activated, form of caspase 3 could be observed early after treatment with staurosporine in infected cells (Figure 2C), showing that hantavirus infection does not completely block initial steps in the process leading to apoptosis induced by staurosporine. However, in line with the observed inhibition of apoptosis in infected cells (Figure 2A–B), significantly lower levels of the cleaved, activated, form of caspase 3 were observed in infected, compared to uninfected, cells (Figure 2C), suggesting that hantavirus can at least partially inhibit activation of caspase 3. As expected, this was mirrored by significantly lower levels of caspase 3-activity (Figure 2D), and less caspase 3-cleaved PARP (Figure 2E) in infected, compared to uninfected, staurosporine-treated cells.

### Hantavirus nucleocapsid protein inhibits the activity of caspase 3

**Figure 3:**
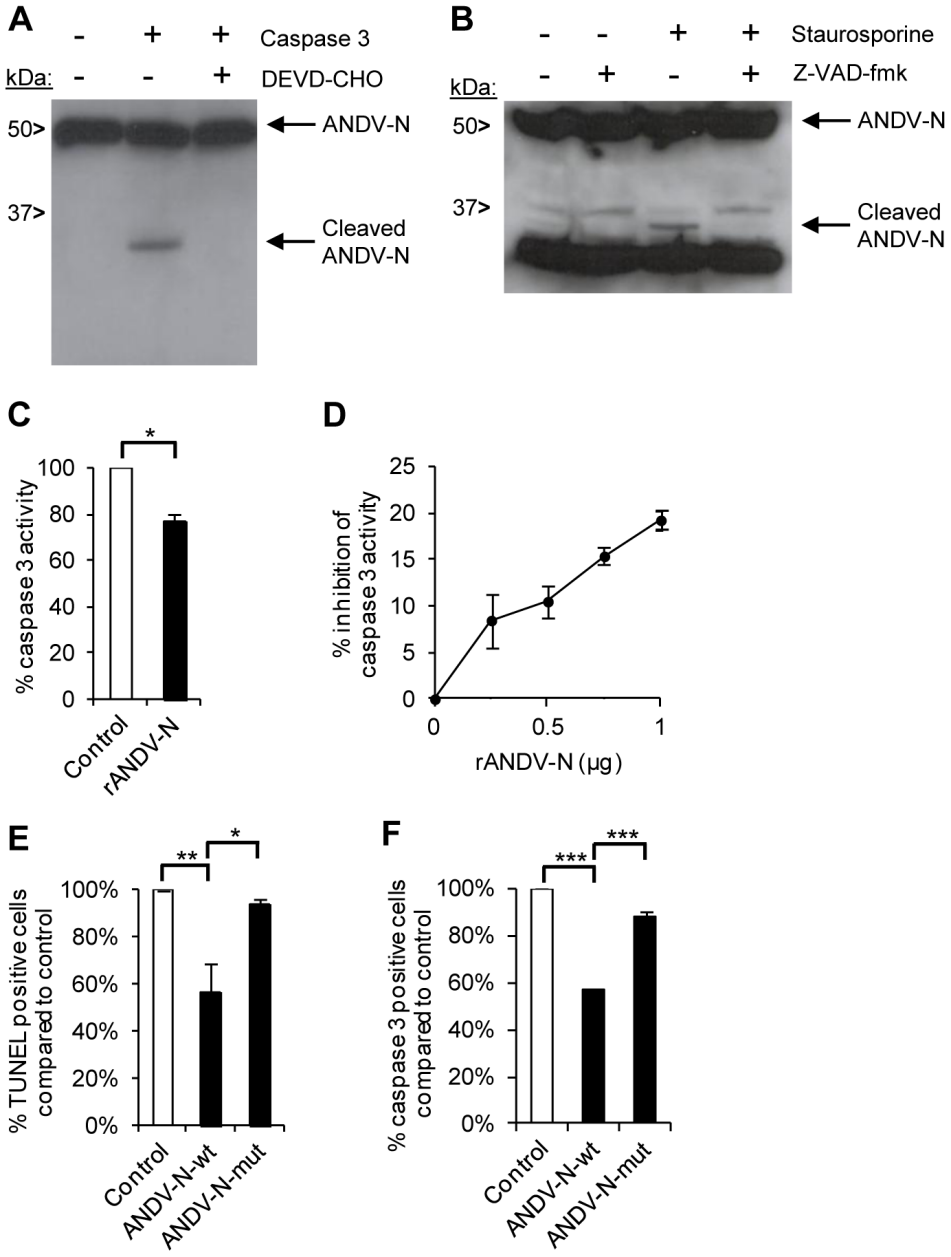
ANDV nucleocapsid protein inhibits caspase 3-activity. Analysis of possible interactions between ANDV nucleocapsid protein and caspase 3. (**A**) Western blot analyses showing a cleaved fragment of the ANDV nucleocapsid protein (ANDV-N) after incubation with recombinant caspase 3 in the presence or absence of the caspase 3-inhibitor DEVD-CHO. Full-length and cleaved ANDV-N was visualized with the mAb 7B3/F7. One representative experiment out of three is shown. (**B**) ANDV-infected A549 cells were left untreated or treated with staurosporine, in the presence or absence of the caspase-inhibitor Z-VAD-fmk. Lysed cells were then subjected to Western blot analyses to visualize cleavage of the viral ANDV nucleocapsid protein (ANDV-N) after staurosporine-treatment. Full-length and cleaved ANDV-N was visualized with the mAb 7B3/F7. One representative experiment out of three is shown. (**C**) Caspase 3-activity was measured after pre-incubation of recombinant caspase 3 with recombinant ANDV nucleocapsid protein (rANDV-N) or with control protein (rDHFR) for 30 minutes. Level of caspase 3-activity after co-incubation with rDHFRS represent maximal caspase 3 activity. Level of caspase 3-activity after co-incubation of caspase 3 with rANDV-N was compared with caspase 3-activity after co-incubation with rDHFRS. Data shown are mean ± SEM of three independent experiments carried out in duplicate. Two-tailed Student's t test was used for statistical evaluation; * p<0.05. (**D**) Caspase 3-activity after pre-incubation for 30 minutes with increasing amount of recombinant ANDV nucleocapsid protein (rANDV-N). Data shown are mean ± SEM of three independent experiments carried out in duplicate. One-way ANOVA was used for statistical evaluation; p<0.05 for 1 µg versus 0.5 µg and for 1 µg versus 0.25 µg. (**E**) Percentage of TUNEL-positive A549 cells transfected with PCMV-bios (control), PCMV-bios-ANDV-N-wt (ANDV-N-wt), or PCMV-bios-ANDV-N Asp285 to Ala285 mutant (ANDV-N-mut) for 20 hours, and then treated with staurosporine for four hours. TUNEL-positive cells were identified using fluorescence microscopy. Staurosporine-treated cells transfected with empty plasmid represent maximal level of TUNEL-positive cells. Data shown are mean ± SEM of three independent experiments carried out in duplicate. One-way ANOVA was used for statistical evaluation; * p<0.05; ** p<0.01. N, nucleocapsid protein; wt, wild-type; mut, mutated. (**F**) Percentage of caspase 3-positive A549 cells transfected with PCMV-bios (control), PCMV-bios-ANDV-N-wt (ANDV-N-wt), or PCMV-bios-ANDV-N Asp285 to Ala285 mutant (ANDV-N-mut) for 20 hours, and then treated with staurosporine for four hours. Caspase 3-positive cells, positive for FLICA-staining, were identified using fluorescence microscopy. Staurosporine-treated cells transfected with empty plasmid represent maximal level of caspase 3-positive cells. Data shown are mean ± SEM of three independent experiments carried out in duplicate. One-way ANOVA was used for statistical evaluation; *** p<0.001. N, nucleocapsid protein; wt, wild-type; mut, mutated.

Certain viruses encode proteins that antagonize caspase enzyme-function either by interacting with their active site (direct inhibition), leading to cleavage of the viral protein and inhibition of the caspase, or by acting as competitive inhibitors of proteins needed for caspase activation (indirect inhibition) [20]. Thus, we hypothesized that inhibition of caspase 3 might be one mechanism used by hantaviruses to prevent infected cells from undergoing apoptosis. Although the canonical caspase 3 cleavage-site DEVD is not present in any hantavirus protein, initial *in silico* analysis revealed that the nucleocapsid protein, found at high levels in the cytoplasm of infected cells [4], contains putative cleavage sites for caspase 3 (e.g., see Figure S1 for *in silico* data of ANDV nucleocapsid protein). Indeed, in support of this, co-incubation of ANDV nucleocapsid proteins with recombinant active caspase 3 resulted in formation of a novel nucleocapsid protein-fragment with a molecular mass of approximately 35 kDa (Figure 3A). To test if the nucleocapsid protein was cleaved in a similar manner in hantavirus-infected cells, these cells were treated with staurosporine followed by analyses of the nucleocapsid protein cleavage-pattern. Interestingly, a fragment of the nucleocapsid protein with a molecular mass equivalent to that observed in Figure 3A was detected also in staurosporine-treated infected cells (Figure 3B). Importantly, this specific fragment was not observed in staurosporine-treated cells in the presence of the caspase-inhibitor Z-VAD (Figure 3B), suggesting that the nucleocapsid protein is a natural caspase 3-target. This finding prompted us to seek for the ANDV nucleocapsid caspase 3-cleavage site. N-terminal sequencing of the caspase 3-cleaved ANDV nucleocapsid protein fragments revealed that the cleavage occurred after Asp_285_, one of the *in silico*-predicted caspase 3-cleavage sites (Figure S1), and in line with the observed approximately 35 kDa product observed in Figure 3A and 3B. Furthermore, recombinant caspase 3 was also able to cleave the nucleocapsid protein in lysates from cells transfected with a plasmid expressing the wild-type ANDV nucleocapsid protein, but not in lysates from cells transfected with a plasmid expressing an Asp_285_ to Ala_285_-mutated variant of the ANDV nucleocapsid protein (data not shown), verifying the presence of the identified caspase 3-cleavage site. We next analyzed if the nucleocapsid protein could inhibit caspase 3. Active recombinant caspase 3 was pre-incubated with recombinant ANDV nucleocapsid protein, or with a control protein, and caspase 3-activity was then measured. Pre-incubation of caspase 3 with ANDV nucleocapsid protein, as compared to the control protein, did indeed result in a measurable inhibition of caspase 3-activity (Figure 3C–D). Finally, to confirm that the identified caspase 3-cleavage site in the nucleocapsid protein was responsible for inhibiting caspase 3-activity, we transfected A549 cells either with wild-type ANDV nucleocapsid protein or with the Asp_285_ to Ala_285_-mutated variant, and subsequently exposed the cells to staurosporine. Transfection of cells with wild-type ANDV nucleocapsid protein rendered cells significantly less susceptible to apoptosis induction (Figure 3E) and displayed reduced caspase 3 activity (Figure 3F). The apoptosis-resistance as well as the caspase 3-activity was reverted when the caspase 3-cleavage site was mutated (Figure 3E–F).

Taken together, the results show that hantavirus nucleocapsid protein significantly inhibits apoptosis and as such the protein represents a new viral caspase 3-inhibitor, similar to baculovirus p35 and p49 and poxvirus cytokine response modifier A (CrmA) [20].

### Hantavirus nucleocapsid protein inhibits the activity of granzyme B

**Figure 4:**
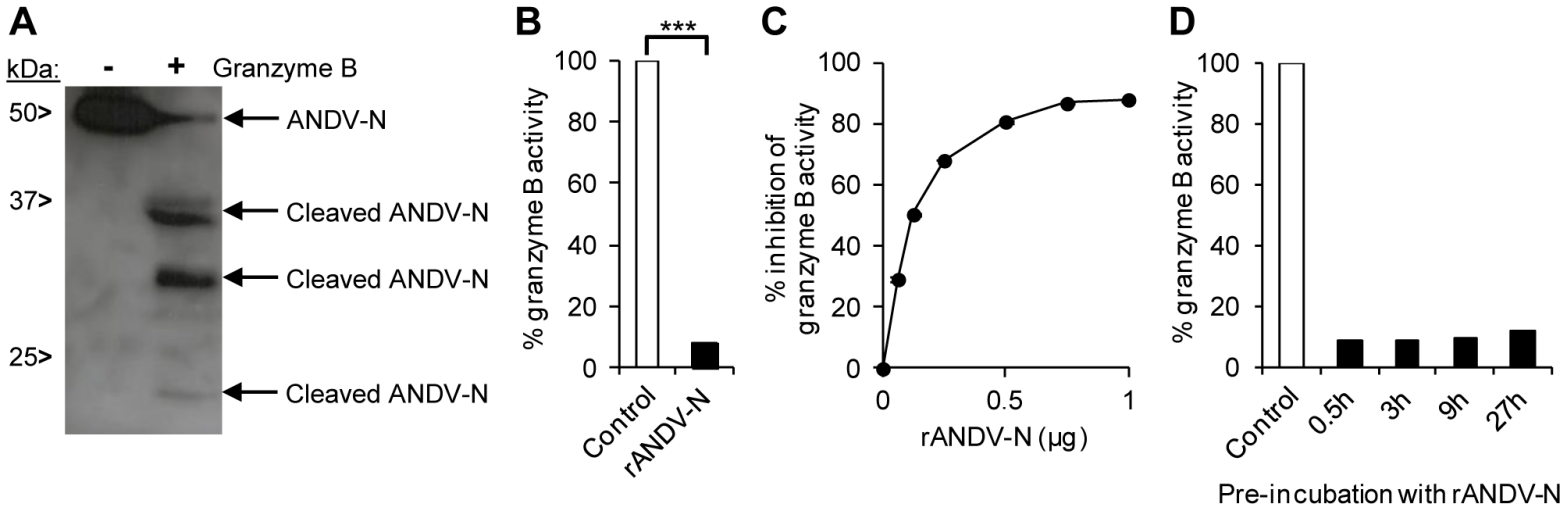
ANDV nucleocapsid protein inhibits granzyme B-activity. Analysis of possible interactions between ANDV nucleocapsid protein and granzyme B. (**A**) Western blot analyses showing cleaved fragments of the ANDV nucleocapsid protein (ANDV-N) after incubation with recombinant granzyme B. Full-length and cleaved ANDV-N was visualized with the mAb 7B3/F7. One representative experiment out of three is shown. (**B**) Granzyme B-activity was measured after pre-incubation of recombinant granzyme B with recombinant ANDV nucleocapsid protein (rANDV-N) for 30 minutes. Results were compared to granzyme B-activity for granzyme B pre-incubated with control protein (rDHFR). Activity after co-incubation with rDHFRS represents maximal granzyme B-activity. Data shown are mean ± SEM of three independent experiments carried out in duplicate. Two-tailed Student's *t* test was used for statistical evaluation; *** p<0.001. (**C**) Granzyme B-activity after pre-incubation for 30 minutes with increasing amount of recombinant ANDV nucleocapsid protein (rANDV-N). Data shown are mean ± SEM of three independent experiments carried out in duplicate. One-way ANOVA was used for statistical evaluation: except for 1 µg versus 0.75 µg (non significant), p<0.001 for all other comparisons. (**D**) Granzyme B-activity after pre-incubation for 0.5–27 hours of recombinant granzyme B with recombinant ANDV nucleocapsid protein (rANDV-N) compared to control protein (rDHFR). Data shown are from one experiment carried out in duplicate.

Cytotoxic lymphocytes use the cytotoxic granule-pathway to induce apoptosis in virus-infected cells. During this process, granzyme B activates caspase 3. However, granzyme B might also promote cell death independent of caspase 3 [21], suggesting that inhibition of caspase 3 alone may not fully account for the failure of NK cells to kill hantavirus-infected cells. Two viral proteins, the 100K assembly protein of human adenovirus type 5 (Ad5-100K) and poxvirus CrmA, were previously shown to inhibit granzyme B [20]. Interestingly, CrmA inhibited both caspase 3 and granzyme B [20], [22]–[24]. These reports led us to investigate if the hantavirus nucleocapsid protein could represent a previously unknown virus-encoded protein capable of also inhibiting granzyme B. Incubation of ANDV nucleocapsid protein with recombinant active granzyme B resulted in cleavage of the nucleocapsid protein into at least three fragments (Figure 4A), showing that these two proteins can interact. As evident by the enzyme-specific cleavage patterns (Figure S2), caspase 3 and granzyme B targeted different sites in the nucleocapsid protein, showing that the nucleocapsid protein likely contains multiple enzyme-specific cleavage sites.

Interestingly, pre-incubation with recombinant ANDV nucleocapsid protein resulted, strikingly, in an almost complete inhibition of granzyme B activity (Figure 4B and C). Although the inhibitory effect somewhat decreased over time, almost 90% inhibition remained even after 27 hours of pre-incubation (Figure 4D), suggesting that the nucleocapsid protein is a potent viral granzyme B-inhibitor.

### Hantavirus inhibits NK cell-mediated activation of caspase 3 in endothelial cells

**Figure 5:**
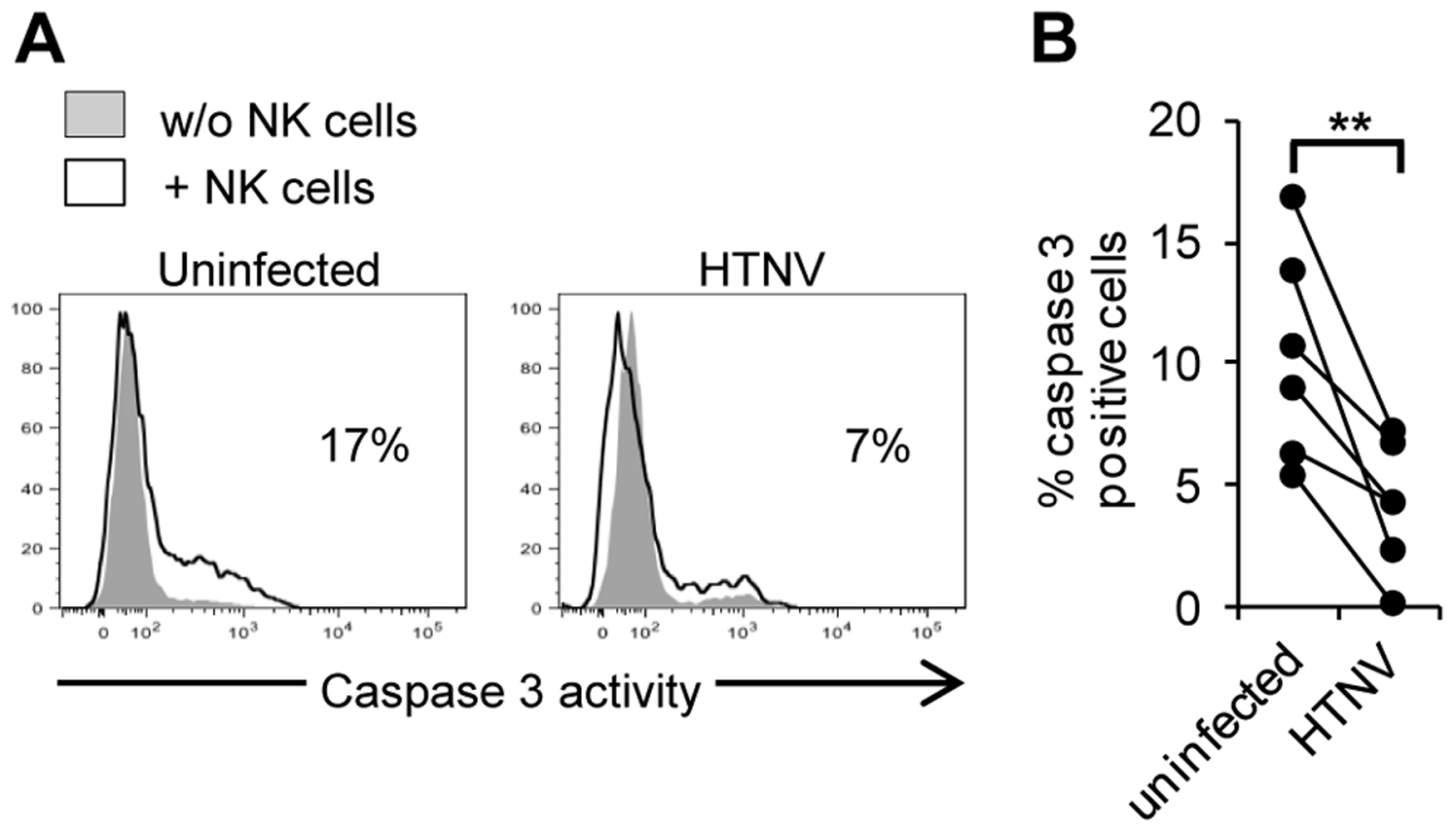
Hantavirus-infection inhibits NK cell-mediated activation of caspase 3 in endothelial cells. Prevention of caspase 3 activation in infected cells exposed to IL-2-activated NK cells. (**A**) Representative flow cytometry histogram showing cellular caspase 3-activity in uninfected and HTNV-infected HLA class I blocked endothelial cells with and without co-incubation with IL-2-activated NK cells. Data shown is one representative donor out of six. (**B**) Percentage of caspase 3-positive uninfected and HTNV-infected endothelial cells after co-incubation with IL-2-activated NK cells analyzed by flow cytometry. Data shown represent two independent experiments from six donors. Two-tailed Student's *t* test was used for statistical evaluation; ** p<0.01.

As mentioned, NK cells normally kill infected cells through cytotoxic granule-mediated induction of apoptosis, involving granzyme B-mediated activation of caspase 3 [13], [14], [20]. To test if hantavirus inhibited NK cell-activation of caspase 3, we exposed uninfected and HTNV-infected endothelial cells to IL-2 activated NK cells for two hours, and then analyzed caspase 3-activity in the endothelial cells. Indeed, significantly fewer infected, than uninfected, endothelial cells were positive for active caspase 3 (Fig. 5A–B), showing that NK cells fail to activate caspase 3 in hantavirus-infected cells.

## Discussion

Cytotoxic lymphocytes play important roles in host responses against virus-infected cells, mainly via their unique ability to kill infected cells through cytotoxic granule-mediated induction of apoptosis [13], [14], [20]. Consequently, some viruses have evolved evasion strategies specifically targeting this pathway of apoptosis-induction [14], [20], [25]–[28]. However, only two viruses have previously been reported to encode proteins that specifically inhibit granzyme B-activity. The human adenovirus type 5 encoded Ad5-100K specifically inhibits granzyme B, whereas the poxvirus CrmA inhibits granzyme B, caspase 3 and other caspases [20], [22]–[24], [29]. Adenovirus and poxvirus are DNA-viruses expressing 20 to 200 viral proteins; in contrast hantaviruses are small RNA viruses encoding only four structural proteins [5]. In this regard, it is striking that hantaviruses harbor such a specific viral evasion strategy. It may suggest that hantavirus-mediated inhibition of granzyme B, and hence of cytotoxic lymphocyte-mediated killing of infected cells, is a crucial component in the life cycle of hantavirus infection. In addition to activating apoptosis via caspase 3, granzyme B can induce cell death in caspase 3-independent manners, e.g., via cleavage of the Rho-associated coiled coil-containing protein kinase II [30], and might also possess direct antiviral functions [28], [31]–[34]. Therefore, strong suppression of granzyme B-activity is likely needed for efficient inhibition of its functions.

Some of the granzyme B that reaches the infected target cell are found in the nucleus [14]. This translocation of granzyme B from the cytoplasm to the nucleus is believed to involve importin α [32], [35]. The nucleocapsid protein of some hantaviruses, like HTNV, can bind importin α and prevent its shuttling capacity [36]. It has been speculated that other hantaviruses, like ANDV, might also have this potential [37]. Hence, the nucleocapsid protein might both directly inhibit the function of granzyme B but also indirectly by inhibiting its functions in the nucleus [14].

CD8 T cells have been shown to be able to kill target cells that express hantavirus nucleocapsid protein [38]–[40]. In these studies the nucleocapsid protein was expressed in the target cells using viral vectors. However, to the best of our knowledge, it has never been shown that CD8 T cells can efficiently kill hantavirus-infected target cells. Like NK cells, CD8 T cells primarily rely on the cytotoxic granule-dependent pathway to kill virus-infected cells [13], [14], [41]. Here, we observed that NK cells are largely incapable of killing hantavirus-infected endothelial cells, strongly suggesting that CD8 T cells will not be able to efficiently kill hantavirus-infected endothelial cells either. This raises the question why cells expressing the nucleocapsid protein were not protected against CD8 T cells in past studies [38]–[40]. It can be speculated (i) that the amount, and (ii) that the localization of the nucleocapsid protein might differ between transfected and infected cells, and furthermore (iii) that other hantavirus and/or cellular factors might be needed for efficient inhibition of cytotoxic lymphocyte-mediated apoptosis by providing hereto unknown anti-apoptotic mechanism(s) that act in parallel to, or in combination with, the nucleocapsid protein.

The findings that hantavirus inhibits staurosporine-induced apoptosis and that the nucleocapsid protein inhibited caspase 3-activity and apoptosis suggest that hantavirus can interfere with induction of apoptosis in general. However, the observed caspase 3-inhibition may not by itself explain the strong anti-apoptotic effect observed in staurosporine-exposed hantavirus-infected cells. It is possible that hantavirus might affect induction of apoptosis also upstream of caspase 3. Possible additional apoptosis-inducing pathway(s) that hantaviruses may inhibit remains to be identified. This is also true for the observed protection against NK cells; while inhibition of granzyme B and caspase 3 is likely to explain a significant part of the hantavirus-mediated protection of infected cells against NK cells, it is possible that also other, hereto unknown, mechanisms are involved. Furthermore, an interesting question is if different hantaviruses use the same mechanisms to inhibit apoptosis. We observed strong inhibition against staurosporine-induced apoptosis and against cytotoxic lymphocyte-killing both in ANDV and HTNV-infected cells, showing that they share the capability to inhibit apoptosis. However, the caspase 3-cleavage site, DLID_285_, detected in ANDV nucleocapsid protein is not conserved among hantaviruses, and while the HTNV nucleocapsid protein, as well as nucleocapsid proteins encoded by other hantaviruses, also contain several *in silico* predicted caspase 3 and granzyme B-cleavage sites (our unpublished observation), it remains to be shown if they represent functional cleavage sites and if they are involved in inhibition of caspase 3 and/or granzyme B.

We have previously observed increased levels of caspase-cleaved cytokeratin 18, a specific marker for epithelial cell apoptosis, during the acute phase of HFRS [42]. While endothelial cells are the main targets for hantavirus, epithelial cells are also infected. The present study demonstrates that hantavirus efficiently inhibits apoptosis also in infected epithelial cells. Interestingly, in this context, it has previously been reported that hantavirus induces apoptosis in uninfected, but rarely in infected, cells [43]. It can be speculated that hantavirus-infection may cause increased levels of bystander apoptosis in surrounding uninfected cells also in patients, and that this may account for the observed increased epithelial cell apoptosis. If this phenomenon also occurs in endothelial cells, and if it is involved in causing the increased vascular permeability during HFRS/HCPS, remains to be investigated.

Vascular leakage is a hallmark of hemorrhagic fevers [3]. However, the mechanisms behind hantavirus infection-associated increased vascular permeability and clinical symptoms of HFRS/HCPS are not well understood. As eluded to above, a matter of discussion has been whether hantaviruses directly are involved in the pathogenesis and/or if immunologic components, i.e., cytotoxic lymphocytes, are responsible [1], [4]–[6]. In the latter case, both their cytotoxic as well as their cytokine producing capacity have been suggested to be associated with pathogenesis. Given the remarkably strong cytotoxic lymphocyte-responses of hantavirus-infected patients, with up to 50% of their peripheral blood CD8 T cells responding during the acute phase of infection and a unique NK cell-profile including highly elevated levels of activated NK cells present up to 60 days after symptom debut in many of the patients [7]–[9], it has been suggested that cytotoxic lymphocytes are directly involved in the HFRS/HCPS-pathogenesis by causing increased vascular permeability by killing hantavirus-infected endothelial cells [1], [6]. This hypothesis suggests that areas in the endothelial-cell barrier damaged by cytotoxic lymphocyte-mediated killing of hantavirus-infected cells are not efficiently repaired, as there is a shortage of thrombocytes because of the acute thrombocytopenia present in patients, which leads to the observed increased vascular permeability [1], [6]. However, the present finding that hantaviruses efficiently inhibit cytotoxic lymphocyte-mediated killing of infected cells, together with reports that infected endothelial cells in patients are not damaged [4], [10]–[12], and the demonstration that CD8 T cells are not involved in ANDV-induced pathogenesis in a Syrian hamster model [44], suggests that HFRS/HCPS-pathogenesis is not directly caused by cytotoxic lymphocyte-mediated killing of infected cells. On the other hand, it is possible that other cytotoxic lymphocyte functions are involved in HFRS/HCPS-pathogenesis, e.g., their strong ability to secrete inflammatory cytokines that might cause increased vascular permeability by inducing gaps in the endothelial cell layer [6]. Although hantaviruses have no direct cytopathic effects on infected cells [1], [2], [4], a direct role for hantaviruses in causing increased vascular permeability without damaging the infected endothelial cells has also been suggested [4]. Support for such reasoning comes from findings that pathogenic hantaviruses use the β_3_ integrin as receptor for cellular entry, and that hantavirus-β_3_ integrin interactions might contribute to increased vascular permeability [4]. Furthermore, hantaviruses induce VEGF-production [45] and sensitize infected cells to the effects of VEGF [46], suggesting a role for VEGF in causing increased endothelial cell permeability.

The finding that hantavirus nucleocapsid protein is a granzyme B and caspase 3-inhibitor together with the fact that hantaviruses primarily infect endothelial cells (i.e., cells that *per se* do not express granzyme B), suggest that hantaviruses have acquired a specific strategy to prevent cytotoxic lymphocytes from inducing apoptosis in infected endothelial cells. The present findings provide a plausible explanation as to why hantavirus-infected cells readily do not undergo apoptosis in patients, despite the apparently strong cytotoxic lymphocyte responses observed in infected individuals [7]–[12]. It is tempting to speculate that the extraordinary strong cytotoxic lymphocyte responses observed during, and long after, the acute phases of HFRS and HCPS [7]–[9], [40], [47] are in part caused by the inability of effector cells to eliminate hantavirus-infected endothelial cells.

## Materials and Methods

### Cells and viruses

Primary human umbilical vein endothelial cells (HUVECs) were grown according to the manufacturer's (Lonza) instructions using EGM-2 BulletKit (Lonza). Before infection and co-culture experiments with NK cells, HUVECs were grown without supplementing the EGM-2 medium with hydrocortisone. The human lung epithelial cell line A549 (American Type Culture Collection [ATCC] CLL-185) was grown in MEM supplemented with 5% FBS, 100 U/mL of penicillin, and 100 µg/mL of streptomycin (all from Invitrogen). K562 cells (ATCC CCL-243) were grown in complete medium (RPMI 1640, Invitrogen, supplemented with 10% FBS, 100 µg/mL L-glutamin, 100 U/mL penicillin, and 100 µg/mL streptomycin). Buffy coats from healthy human blood donors were obtained from the blood bank at the Karolinska University Hospital Huddinge, Stockholm, Sweden. PBMC were isolated by density centrifugation (Ficoll-Hypaque; GE Healthcare) followed by NK cell isolation (Miltenyi). NK cells were cultured over night in complete medium supplemented with IL-2 (500 U/mL; Proleukin, Chiron Corporation).

ANDV and HTNV were propagated on Vero E6 cells (ATCC Vero C1008) as previously described [48]. For immune fluorescence experiments of cells exposed to staurosporine, cells were infected with multiplicity of infection (MOI) 0.01, resulting in approximately 20% of cells being infected three days post infection. For all other experiments cells were infected with MOI 1; as earlier shown [9] this results in more than 80% of cells being infected at day three post infection (Figure S3). Cells were infected for 3 to 4 days before samples were collected for analyzes.

### Chemicals and proteins

Staurosporine and the pan caspase inhibitor Z-VAD-fmk were from BioVision while the caspase 3-inhibitor DEVD-CHO was from Sigma; all were used at a concentration of 2 µM each. Recombinant ANDV nucleocapsid protein [49] and rDHFR [50] were prepared as described [49], [50] and subsequently subjected to dialysis against PBS. Recombinant active human caspase 3 and granzyme B were from BD Biosciences and Biovision, respectively. The monoclonal antibodies (mAbs) 7A2/D5, 7B3/F7 and 1C12, specific for hantavirus nucleocapsid protein, were used as previously described [49], [51]. MAbs specific for PARP, caspase 3 and calnexin were all from Cell Signaling Technology.

### Co-incubation of NK cells and endothelial cells

Overnight IL-2 stimulated NK cells and infected/uninfected HUVECs were co-incubated at an effector to target ratio of 1∶1, or as specified in the text, at 37°C for 2 hours. Prior to the co-incubation, HLA class I on target cells was blocked using a combination of A6–136 hybridoma (kindly provided by Dr D Pende, Istituto Nazionale per la Ricerca sul Cancro, Genoa, Italy) and Dx17 mAbs (BD Biosciences) for 30 minutes at room temperature.

### Analyses of NK cell degranulation

After co-incubation with target endothelial cells, NK cells were stained with the following antibodies: anti-CD56 PE-Cy7, anti-CD14 V500, anti-CD19 V500, anti-CD107a FITC (all BD Biosciences), and anti-CD3 ECD (Beckman Coulter). A dead cell marker (DCM) was used to exclude dead cells (LIVE/DEAD Fixable Aqua Dead Cell Stain Kit, Invitrogen). Staining was performed for 30 minutes on ice in the dark in PBS containing 2% FBS (FACS buffer), followed by 2 washes with FACS buffer, and subsequent fixation in PBS with 1% PFA for 30 minutes. Cells were acquired on a LSR Fortessa (BD Biosciences) and analyzed with FlowJo software version 9 (Treestar). For the analysis, CD107a expression was evaluated on live NK cells (CD56+CD3−CD14−CD19−DCM−).

### NK cell killing assay

Uninfected and HTNV-infected endothelial cells, and K562 cells, were stained with CFSE (Invitrogen) following the manufacturer's instructions. IL-2 activated NK cells were incubated at different effector to target ratios with 20,000 target endothelial cells or K562 cells (positive control) for 4 hours at 37°C. To analyze NK cell-mediated target cell killing LIVE/DEAD Fixable Aqua Dead Cell Stain Kit was used to stain killed cells: DCM-staining was performed for 30 minutes on ice in the dark in FACS buffer, followed by 2 washes with FACS buffer, and subsequent fixation in PBS with 1% PFA for 30 minutes. Cells were acquired on a LSR Fortessa and analyzed with FlowJo software version 9. For the analysis to detect specific target cells' death, the percentage of DCM-positive cells within CFSE-positive cells was evaluated.

### TUNEL assay

TUNEL assay (Roche) was performed as previously described [52]. After TUNEL reaction, cells were subjected to staining with anti-nucleocapsid protein mAb followed by FITC-conjugated goat anti-mouse IgG (Sigma-Aldrich). Nuclei were counter stained with DAPI (Sigma-Aldrich).

### Immunoblot analysis

Samples to be analyzed were mixed 4∶1 with NuPAGE 4× LDS sample preparation buffer (Invitrogen), supplemented with 2.5% 2-mercaptoethanol, incubated at 96°C for 10 minutes, resolved on 10% NuPAGE Novex Bis-Tris gel (Invitrogen) and transferred to PVDF membranes. Blocking was performed at 4°C for 1 hour in PBS supplemented with 5% nonfat dry milk and 0.2% Tween 20. The membranes were subsequently incubated with mAb for 1 hour at room temperature, followed by the addition of horseradish peroxidase-conjugated anti-mouse IgG (Bio-Rad). Chemiluminescence substrate (ECL Plus Western blotting detection kit, GE Healthcare) was used following the manufacturer's protocol. Membranes were stripped in stripping buffer (10 mM 2-mercaptoethanol, 2% SDS and 62.5 mM TRIS-HCL [pH 6.7]). Samples from staurosporine-treated cells were homogenized in lysis buffer (150 mM NaCl, 2 mM EDTA, 1% NP-40, and 50 mM Tris [pH 7.6]) supplemented with complete protease inhibitor cocktail minitablets (Roche Diagnostics) prior to analyzes. Band densitometry was analyzed with the program Image J (NIH).

### Caspase 3 and granzyme B activity assays

Caspase 3 and granzyme B activities were analyzed using specific activity assays according to the manufacturers' instructions (Sigma for caspase 3, Calbiochem for granzyme B). When analyzing cellular caspase 3 activity after staurosporine-treatment, total concentrations of cellular protein in samples were analyzed by a Bradford assay (Biorad) according to the manufacturers' instructions as internal control. To analyze nucleocapsid protein-specific inhibition of caspase 3 and granzyme B activity, recombinant human caspase 3 (0.1 µg) or active recombinant granzyme B (0.1 µg) was incubated with 1 µg recombinant ANDV nucleocapsid protein or 1 µg rDHFR as control, for 30 minutes or as stated in the text. When analyzing the effect different concentrations of the nucleocapsid protein had on caspase 3 and granzyme B activity, rDHFR were added to the samples to adjust the total amount of rANDV nucleocapsid protein+rDHFR proteins in all samples to 1 µg.

Cellular caspase 3-activity was also analyzed using Fluorescent-Labeled Inhibitors of Caspases (FLICA) according to the manufacturer's (Immunochemistry Technologies) instructions. Briefly, target endothelial cells were co-incubated with IL-2-activated NK cells at an effector to target ratio of 1∶1 at 37°C for two hours, where the FLICA-probe FAM-DEVD-fmk was added for the last hour. Cells were subsequently stained with DCM, acquired on a LSR Fortessa, and analyzed with FlowJo software version 9. Specific NK cell-induced activation of caspase 3-activity in target cells was calculated as percent of total active caspase 3-positive target cells minus percent of active caspase 3 positive endothelial cells not exposed to NK cells.

### Nucleocapsid protein cleavage assays

Heat-inactivated ANDV (10 minutes at 96°C) was incubated with either active recombinant human caspase 3 or granzyme B in reaction buffer (BioVision) at 37°C overnight and then subjected to immunoblotting for analysis of cleaved nucleocapsid protein.

Edman degradation was performed by Alphalyse, Denmark.

### Expression of ANDV nucleocapsid protein

Empty plasmid (PCMV-bios), and plasmids expressing wild-type ANDV nucleocapsid protein (PCMV-bios-ANDV-N-wt) and Asp_285_ to Ala_285_ mutated ANDV nucleocapsid protein (PCMV-bios-ANDV-mut, constructed by Genscript, USA) were prepared with EndoFree plasmid maxi kit (Qiagen) and transfected into A549 cells using Lipofectamine LTX (LifeSciences) according to the manufacturer's instructions.

### Statistical methods

All data were analyzed using Prism (GraphPad Software). Values are represented as mean ± SEM. Statistical analysis were performed using one-way or two-way ANOVA, followed by posthoc tests, or by two-tailed Student's *t* test. P-values<0.05 were considered significant, where * p<0.05; ** p<0.01 and *** p<0.001.

### Accession numbers

Recombinant ANDV nucleocapsid protein used in this study: AY228237.1

Hantaviruses used in this study: ANDV; AF291702.1, AF291703, AF291704.5, HTNV; M14626, M14627, X55901.

## Supporting Information

Figure S1 **ANDV nucleocapsid protein contains putative caspase 3-cleavage sites.** ANDV nucleocapsid protein has several *in silico*-predicted possible caspase 3-cleavage sites (http://casbase.org/casvm/server/index.html). The predicted cleavage sites are marked in bold and the target aspartic acids are marked in red.
(TIF)

Figure S2 **Hantavirus nucleocapsid protein is cleaved by caspase 3 and granzyme B at enzyme-specific sites.** Western blot analyses of ANDV nucleocapsid protein (ANDV-N) after incubation with recombinant active granzyme B or caspase 3. Full-length and cleaved ANDV-N was visualized with the mAb 1C12.
(TIF)

Figure S3 **Level of ANDV and HTNV infected endothelial cells.** Percentage of ANDV and HTNV-infected endothelial cells three days post infection. Cells were stained for hantavirus nucleocapsid protein with the mAb 7B3/F7 for ANDV and the mAb 7A2/D5 for HTNV, respectively, and DAPI for nuclear staining. Levels of infected cells were then quantified using fluorescence microscopy. Data shown represent mean ± SEM from 3 independent experiments. Two-tailed Student's t test was used for statistical evaluation; N.S.; not significant.
(TIF)
