# Supplementary figures and images for: Hantavirus-infection Confers Resistance to Cytotoxic Lymphocyte-Mediated Apoptosis
Source: PLoS Pathog. 2013 Mar 28;9(3):e1003272. doi: 10.1371/journal.ppat.1003272 (PMC3610645; doi:10.1371/journal.ppat.1003272)

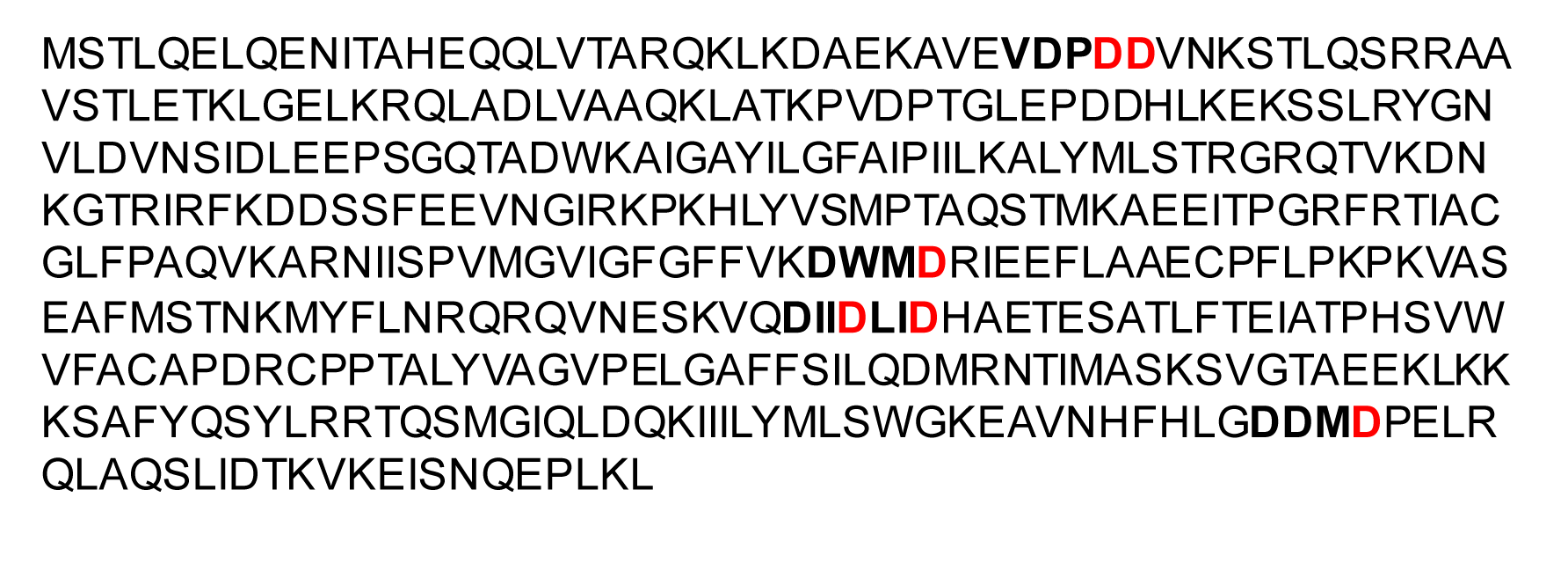

Supplement: Figure S1 — ANDV nucleocapsid protein contains putative caspase 3-cleavage sites. ANDV nucleocapsid protein has several in silico-predicted possible caspase 3-cleavage sites (http://casbase.org/casvm/server/index.html). The predicted cleavage sites are marked in bold and the target aspartic acids are marked in red. (TIF) [file ppat.1003272.s001.tif]

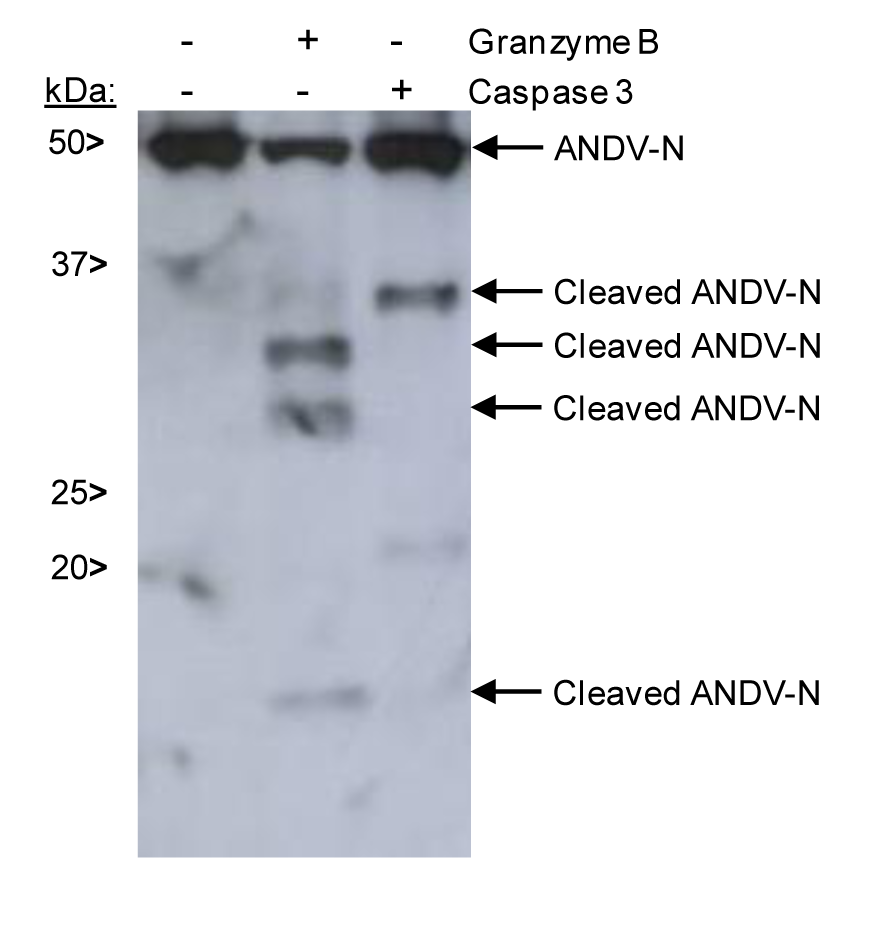

Supplement: Figure S2 — Hantavirus nucleocapsid protein is cleaved by caspase 3 and granzyme B at enzyme-specific sites. Western blot analyses of ANDV nucleocapsid protein (ANDV-N) after incubation with recombinant active granzyme B or caspase 3. Full-length and cleaved ANDV-N was visualized with the mAb 1C12. (TIF) [file ppat.1003272.s002.tif]

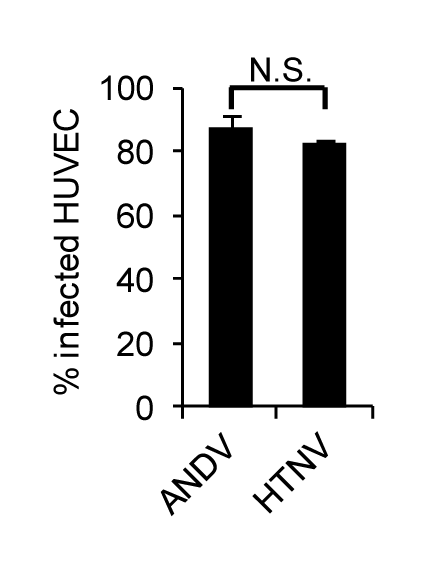

Supplement: Figure S3 — Level of ANDV and HTNV infected endothelial cells. Percentage of ANDV and HTNV-infected endothelial cells three days post infection. Cells were stained for hantavirus nucleocapsid protein with the mAb 7B3/F7 for ANDV and the mAb 7A2/D5 for HTNV, respectively, and DAPI for nuclear staining. Levels of infected cells were then quantified using fluorescence microscopy. Data shown represent mean ± SEM from 3 independent experiments. Two-tailed Student's t test was used for statistical evaluation; N.S.; not significant. (TIF) [file ppat.1003272.s003.tif]
